# Molecular Dynamics Simulation of the Cu_3_Sn/Cu Interfacial Diffusion Mechanism under Electrothermal Coupling

**DOI:** 10.3390/ma16247507

**Published:** 2023-12-05

**Authors:** Zhiwei He, Xin Lan, Lezhou Li, Yong Cheng

**Affiliations:** 1 School of Energy and Power Engineering, Shandong Univerisity, Jinan 250061, China; hezhiwei_sdu@163.com (Z.H.); lilezhou2021@163.com (L.L.); cysgd@sdu.edu.cn (Y.C.); 2 Yuanshan (Jinan) Electric Co., Ltd., Jinan 250021, China

**Keywords:** Cu_3_Sn/Cu interface, molecular simulation, electric field intensity, diffusion activation energy, diffusion mechanism

## Abstract

With the increasing power density of electronic devices, solder joints are prone to electromigration under high currents, which results in a significant threat to reliability. In this study, the molecular dynamics method is used to study the diffusion mechanism of the Cu_3_Sn/Cu interface under the action of electrothermal coupling. The results show that the diffusion activation energy decreases with an increase in electric field intensity, accelerating the diffusion of the Cu_3_Sn/Cu interface. Furthermore, it is noted that the abrupt change in the vacancy–time curve lags behind that of the mean square displacement curve, which depicts that the responses of the vacancies are driven by the electric field. The vacancy-responsive diffusion mechanism of the Cu_3_Sn/Cu interface is proposed. The atoms around the interface in the electric field get rid of the shackles of the neighboring atoms easily. The vacancy concentration increases as the atoms leave the equilibrium position, which accelerates the movement of vacancies and enhances the diffusion of the Cu_3_Sn/Cu interface.

## 1. Introduction

**Figure 1:**
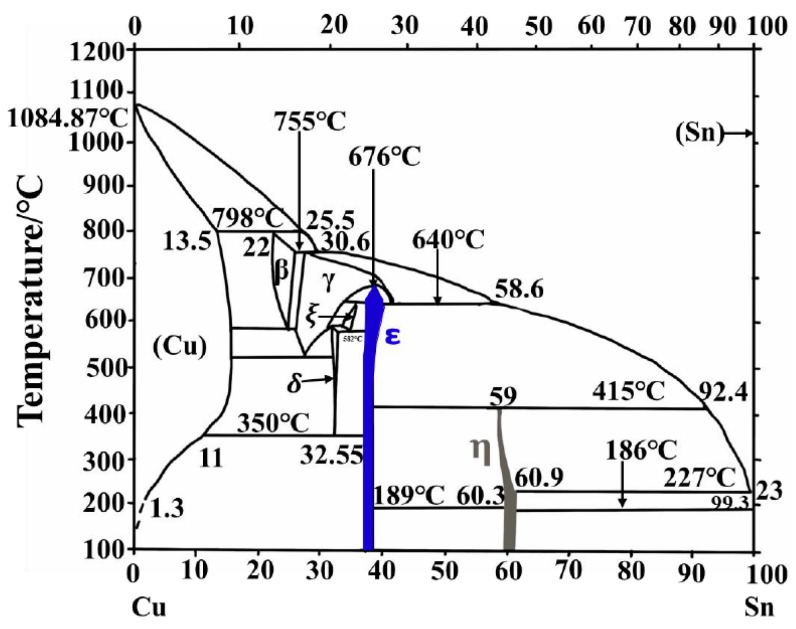
Sn-Cu binary phase diagram [3].

Electronic devices are progressing towards miniaturization, increased integration, and expanded functionality. The current density and thermal loads increase significantly with the development of microelectronic devices [1]. Under high temperature and current density conditions, severe electromigration will occur at the interface [2]. The Sn atoms of the Sn-Ag-Cu (SAC) solder and the Cu atoms of the Cu pad combine easily to form intermetallic compounds (IMC) under electromigration and thermal aging conditions. Figure 1 shows the equilibrium phase diagram of Cu-Sn binary alloy [3], in which Cu_3_Sn (ε phase) and Cu_6_Sn_5_ (η phase) are the two most important intermetallic compounds. When the temperature is higher than 186 °C, the η phase will transform from a long-range-ordered intermetallic compound to a short-range-ordered intermetallic compound. When the mass ratio of Sn is 99.3%, a eutectic reaction will occur at 227 °C [4]. It can be seen from Figure 1 that for a mixture of Cu_6_Sn_5_ and Sn, in which Sn accounts for 61–100% of the total material mass ratio, the melting point is 227 °C. For a mixture of Cu_3_Sn and Cu_6_Sn_5_, with Sn accounting for 38–61% of the total material mass, the melting point is 415 °C. Therefore, when the total material mass occupied by Sn is reduced to less than 61%, the melting point of the alloy will jump from 227 °C to 415 °C. That is, under lower temperature conditions, Cu-Sn achieves bonding and generates an alloy with a higher melting point [5]. However, the formation of IMC normally leads to Kirkendall voids and cracks around the interface or within the solder joint, and the resulting fatigue failures are more pronounced under cyclic loadings [6]. Additionally, the creation of voids is expedited near the Cu_3_Sn/Cu interface because of the substantial variance in diffusion rates for Cu and Sn atoms under external fields. During subsequent diffusion, these voids coalesce and enlarge, resulting in the deterioration of mechanical integrity. Consequently, this elevates the susceptibility to failure in electronic devices. Therefore, the study of the interfacial diffusion behavior of Cu_3_Sn/Cu has attracted widespread attention.

It has been experimentally proven that an electric field affects the diffusion behavior of the atoms around the interface of solder joints [7,8,9]. Fu et al. [10] studied the electromigration behaviors of lead-free solder. The experimental results reveal that the orientation of tin-based solder joints atop copper pillars, under the combined effects of electron wind forces and thermal gradients, markedly impacts the level of electromigration-induced deterioration. Liu et al. [11] experimentally investigated the evolution of the microstructure of Cu/Cu_3_Sn/Cu interfaces by applying electric current to the solder samples. Cellular voids were found in the middle of the solder joints. The voids grew with the duration of electrification, and the formation time of voids and defects was greatly shortened with the increase in current density. Wang et al. [12,13] studied the effects of electric field annealing on the diffusion of the Cu/Ta/Si interface through experiments. The research revealed that the application of an external electric field expedites the penetration of Cu into the Ta layer. The acceleration effect is mainly attributed to the disturbance of the electrical state of the interface and the internal defects of the grains. Increasing the annealing temperature enhances the electric field’s impact on atomic diffusion. Zhang et al. [14] studied the IMC evolution and whisker growth of Sn-0.3Ag-0.7Cu (SAC0307) microsolder joints. Numerical and experimental investigations were carried out to explore the coupling effects between thermal, mechanical, and electrical phenomena. The results revealed that atomic diffusion was most prominently affected by the electric field intensity. Li et al. [15] studied the change in the Al/Cu interface structure of the solder joint under a DC electric field. The evolution mechanism of IMC under the current loading was analyzed. The experimental results show that heightened IMC thickness at the interface can be attributed to the stimulation of the electric field, variations in concentration, and temperature disparities, all of which influence the diffusion and movement of Al and Cu atoms throughout the electromigration. The experimental part presents more macroscopic results. It is difficult to observe the interface diffusion process at the atomic scale and obtain the interface diffusion mechanism. 

At the micro–nano scale, molecular dynamics (MD) has emerged as a potent technique for analyzing the diffusion of heterogeneous atoms. It acquires insights into the interaction and progression of atoms in spatial dimensions, solving potential function models pertaining to diverse systems [14].

Cheng et al. [16] calculated the diffusion energy barrier of Cu atoms in Sn crystals under different electric field intensities based on first principles. It illustrated that as the electric field intensity increased, the energy barrier for Cu atom diffusion decreased, thus facilitating the migration of Cu atoms toward Sn atoms. First principles are usually suitable for simulating systems with a small number of atoms, while molecular dynamics is more suitable for studying the dynamic process of large-scale atomic diffusion and revealing its diffusion mechanism. Zhang et al. [17] employed molecular dynamics to simulate the interfacial evolution and whisker growth in copper–tin coatings under electrothermal coupling. The results indicate that heightened electric field intensity and elevated temperature will enhance the migration of copper atoms toward tin atoms, leading to the sequential formation of Cu_6_Sn_5_ and Cu_3_Sn IMCs at the coating interface. Yu et al. [18] examined the Cu_3_Sn/Cu interface’s diffusion behavior across different temperature regimes. The diffusion behavior of atoms at the interface was analyzed and calculated. The results show that the atoms in the copper lattice diffuse at a slower rate, but they can still penetrate the Cu_3_Sn lattice. However, although the atoms in Cu_3_Sn diffuse at a faster rate, it is difficult for them to enter the copper lattice. Guo et al. [19] investigated the atomic diffusion around the Cu_3_Sn/Cu interface in the electric field. The application of an electric field resulted in a notable manifestation of the Kirkendall effect. Enhancing electric field intensity can significantly improve the diffusion coefficient of the Cu_3_Sn side atoms and inhibit the diffusion of Cu crystal side atoms. Yu et al. and Guo et al. studied the diffusion behavior of the Cu_3_Sn/Cu interface based on temperature and electric field intensity, respectively. The interaction and diffusion mechanisms of interface atoms under the coupling effects of the temperature field and electric field have not been considered. 

As aforementioned, the electromigration process performs different behaviors in various temperature fields and electric fields. The electrothermal coupling effect should be further taken into account. Molecular dynamics simulation has not been utilized to investigate the diffusion mechanism at the Cu_3_Sn/Cu interface under electrothermal coupling. The mechanism of the electric field on the diffusion behavior of interfacial atoms in solder joints still needs to be further investigated and revealed. In this study, molecular dynamics was employed to simulate the diffusion of the Cu_3_Sn/Cu interface under electrothermal coupling. The interaction and diffusion mechanisms of atoms around the interface under various temperatures and electric field intensities are explored. The results can provide guidance and reference for the reliability assessment of the electronic assemblies and packaging design of high-power density devices.

## 2. Calculation Principle

### 2.1. Potential Energy Model

Molecular dynamics focuses on a particle ensemble, where the interaction among atoms is depicted through a potential function. It is essential to select the rational potential function and its parameters, which are critical to the accuracy of the simulation. The modified embedded atom method (MEAM) atomic interaction potential can usefully describe the diffusion of metal atoms [20]. Therefore, the MEAM atomic interaction potential is employed in this study to describe the interaction between Cu, Sn, and Cu-Sn atoms. The potential function is shown in Equations (1) and (2).

(1) $$E_{total}=\sum_{i}F_{i}(\rho_{i})+\frac{1}{2}\sum_{i\neq j}\phi_{ij}(R_{ij})$$

(2) $$F_{i}(\rho_{i})=A_{i}E_{i}^{0}\rho_{i}\ln\rho_{i}$$

The electron density, denoted as $\rho_{i}$, is produced by the *j*th atom, with atom *i* located at a distance *R*; $F_{i}(\rho_{i})$ is the embedding energy of the *i*th atom. $\phi_{ij}(R_{ij})$ represents the two-body interaction potential between atom *i* and atom *j*. $E_{i}^{0}$ is the binding energy of atom *i* in the related structure. $A_{i}$ is a structural parameter. The general equations of the electron density potential function and the two-body potential are shown in Equations (3) and (4).

(3) $$\rho =\sum_{j\neq i}f_{i}(r_{ij})+\sum_{\begin{array}{l} j\neq i\\ k\neq i \end{array}}\left[\alpha_{j}^{1}\alpha_{k}^{1}\cos\theta_{jik}-\alpha_{j}^{2}\alpha_{k}^{2}\cos\theta_{jik}\right]f_{j}(r_{ij})f_{k}(r_{ik})$$

(4) $$\phi (r)=\phi \hat{A}•\exp\left[-\gamma \left(\frac{r}{r_{le}}-1\right)\right],r\leq r_{c}$$

where, $f_{j}$ is the electron density of atom *j*. $\theta_{jik}$ represents the angle between atom *i*, *j*, and *k* when atom *i* is at the center. $\alpha_{j}^{1}$ and $\alpha_{j}^{2}$ are constants related to atom *j*. ϕ(r) is the general equation of the two-body potential. $r_{c}$ is the cutoff radius.

The MEAM potential function obtained by Cheng et al. [21] can well describe the interactions between Cu, Sn, and Cu-Sn. The parameters used in this study are referred to in Table 1. The angular screening technique was applied following the approach outlined by Baskes [22]. The value of $C_{\max}$ is established at 2.8 for all element combinations, while $C_{\min}$ equals 0.8 for all element combinations, with the exception being a solitary Cu atom screening two additional Cu atoms, and the rest is $C_{\min}$ = 2.0. The screening parameters are shown in Table 2.

### 2.2. Verlet Algorithm

The Verlet algorithm is one of the most common integration methods in classical mechanics (Newtonian mechanics) and is widely used in molecular dynamics simulation. The Verlet algorithm is expressed in the form of a Taylor expansion as follows [14]:

(5) $$r(t+\delta t)=r(t)+\frac{d}{dt}r(t)\delta t+\frac{1}{2!}\frac{d^{2}}{dt^{2}}r(t){\left(\delta t\right)}^{2}+\cdots$$

(6) $$r(t-\delta t)=r(t)-\frac{d}{dt}r(t)\delta t+\frac{1}{2!}\frac{d^{2}}{dt^{2}}r(t){\left(\delta t\right)}^{2}+\cdots$$

Add Equations (5) and (6) to obtain Equation (7).

(7) $$r(t+\delta t)=-r(t-\delta t)+2r(t)+\frac{d^{2}}{dt^{2}}r(t){\left(\delta t\right)}^{2}$$

Obviously, if the position and acceleration at time *t* and the position at time *t* − *δt* are known, the position at time *t* + *δt* can be calculated by Equation (7).

By subtracting Equations (5) and (6), the calculation formula of velocity at time t can be obtained:

(8) $$v(t)=\frac{1}{2\delta_{t}}[r(t+\delta t)-r(t-\delta t)]$$

If the position at time *t* − *δt* and *t* + *δt* are known, the velocity at time *t* can be calculated by Equation (8). *v*(*t*) is the velocity of the atom at time *t*. *r*(*t*) is the position of the atom at time *t*.

### 2.3. Mean Square Displacement (MSD)

Molecular dynamics with the LAMMPS simulation package can record and output the trajectory of each atom. Analyzing the trajectories of atoms allows for the determination of the mean square displacement (MSD) [23]. According to Einstein’s Law of Diffusion, the diffusion coefficient can be calculated from the MSD of atoms using Equation (9).

(9) $$D=\underset{t\to \infty }{\lim}\frac{L_{MSD}(t)}{6t}=\underset{t\to \infty }{\lim}\frac{\left({\left|r_{i}(t)-r_{i}(0)\right|}^{2}\right)}{6t}$$

where *D* is the diffusion coefficient; $L_{MSD}$ is the mean square displacement; $r_{i}(0)$ represents the initial position of the atom at the initial time; and $r_{i}(t)$ represents the position of atom *i* at time *t*. When the calculation steps are long enough, the slope of the MSD-time curve is 6*D*.

The diffusion coefficient normally follows the Arrhenius equation,

(10) $$D=D_{0}\exp(-\frac{Q}{RT})$$

where *Q* is the diffusion activation energy, *R* is the Boltzmann constant ($1.38\times 10^{-23}$ J/K), and $D_{0}$ is the frequency factor. Equation (10) can be rewritten in the following form:

(11) $$\ln D=\ln D_{0}-\frac{Q}{RT}$$

Equation (11) behaves as a simple linear equation between lnD and $\frac{1}{T}$. Therefore, the value of *Q* can be derived by analyzing the line’s slope, and $\ln D_{0}$ can be determined from the intersection of the line with the lnD axis.

### 2.4. Simulation Process

**Figure 2:**
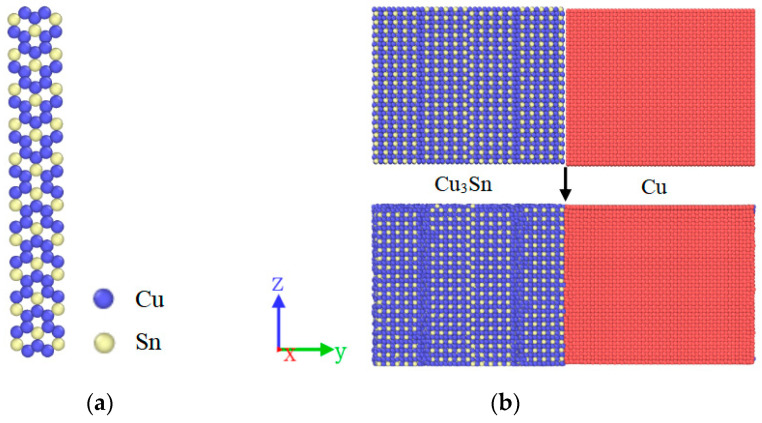
(**a**) Long-period superlattice Cu_3_Sn and (**b**) the Cu_3_Sn-Cu interface structure.

In this research, the diffusion behavior of atoms at the Cu_3_Sn/Cu interface under the action of electrothermal coupling is simulated by the LAMMPS molecular dynamics package. The data are visualized and analyzed by OVITO 3.0.0 software. The Cu_3_Sn compound represents a hexagonal close-packed structure, exhibiting the Cu_3_Ti-type transition-metal IMC. Notably, an ordered form of Cu_3_Sn has been documented as a long-period superlattice alloy [24]. The constructed MD simulation interface structure measures 102 Å × 192 Å × 80 Å in dimensions (X × Y × Z). It considers the Cu (001) surface’s interaction with the Cu_3_Sn (001) surface. The initial models of the long-period superlattice Cu_3_Sn and Cu_3_Sn/Cu interface structures are plotted in Figure 2a,b, respectively. To better study the diffusion behavior of Cu atoms in the Cu_3_Sn/Cu interface, the Cu_3_Sn side Cu atoms are labeled as Cu_1 atoms, and the Cu side Cu atoms are denoted as Cu_2 atoms. Prior to conducting the molecular dynamics simulation, optimization of the structure was performed. The two grains were placed at a distance of 3Å apart from each other in the Y direction. Then, the 5 Å atomic layers on both sides of the model are fixed. Finally, the structure achieved thermal equilibrium using MD at the target temperature. The process is shown in Figure 2b. In the simulation, the Nose-Hoover thermostat was employed to regulate the system temperature, and the numerical equations of motion were solved using the Velocity Verlet algorithm. The initial atomic velocity was randomly assigned based on the Maxwell–Boltzmann distribution. The electric field was applied along the Y axis, and the positive direction of the Y axis was defined as the positive electric field. Periodic boundary conditions are applied in the X and Z directions of the model, and the time step is set to 1 fs.

To better investigate the diffusion process, two monitoring layers were set at a distance of 10 Å from the interface. There are 5358 and 6915 atoms in the Cu_3_Sn side monitoring region and the Cu side monitoring region, respectively. Moreover, the Cu_3_Sn side Cu atoms and Sn atoms are grouped separately, and their MSD values are also output individually. Thereby, it intuitively manifests the variation in the diffusion coefficient of each atom in the system. The system is divided into blocks on the y-axis to output the density/mass values and obtain the corresponding atomic concentration gradient. The plan was operated under the NVT ensemble. The conjugate gradient approach was employed to minimize the system’s energy. Initially, a 30 ps relaxation period was conducted to alleviate atomic overlap and eliminate any implausible structures. This process ultimately guaranteed that the system attained its lowest energy state, forming the initial configuration for diffusion. Next, a 200 ps simulation was executed, recording atom coordinates and displacements at 0.1 ps intervals. The MSD values were extracted against time to obtain the diffusion coefficient.

In this study, the diffusion behavior of the Cu_3_Sn/Cu interface was simulated under various electric fields (0.002, 0.004, and 0.008 V/Å) and temperatures (450 K, 700 K, and 950 K). The internal correlations of concentration gradient, MSD, vacancy concentration, and partial atomic transposition mechanism were systematically investigated. Then, a vacancy response mechanism for Cu_3_Sn/Cu interface diffusion was proposed in conjunction with the diffusion theory.

## 3. Results and Analysis

### 3.1. Effect of Electric Field on the Activation Energy of the Cu_3_Sn/Cu Interface

**Figure 3:**
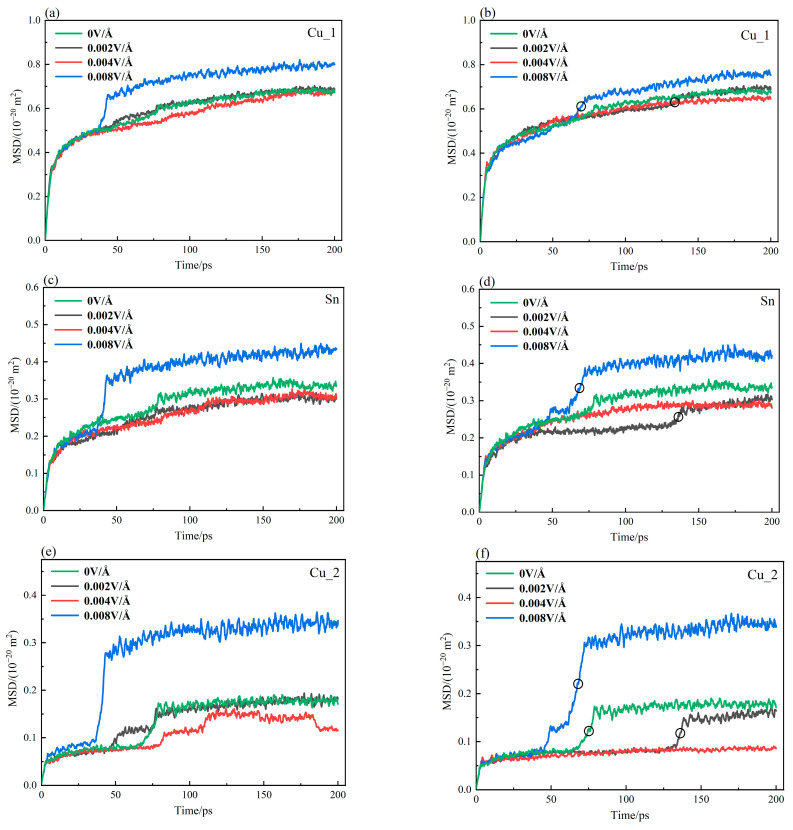
MSD−time curve obtained by the coupled electric field at 450K: (**a**,**b**) Cu_1, (**c**,**d**) Sn, and (**e**,**f**) Cu_2. (The circles in the figure indicate the mutation phase of the curve).

The diffusion coefficient at the Cu_3_Sn/Cu interface was calculated with a temperature of 450K and electric field intensities of 0.002, 0.004, and 0.008 V/Å. The direction of the electric field was also considered in the analysis. The MSD-time curves under a positive electric field for Cu_1, Cu_2, and Sn atoms are plotted in Figure 3a,c,e, respectively. Those for the case under a negative electric field are shown in Figure 3b,d,f, accordingly. In Figure 3a,c,e, the MSD values of the Cu_1 and Sn atoms are larger than those of the Cu_2 atom. This is mainly due to the stronger bond between atoms in the Cu crystal than in the Cu_3_Sn crystal. It consequently leads to a lower probability of the atomic transition in Cu crystal than in Cu_3_Sn crystal, which agrees with the conclusion proposed by Guo et al. [19].

This study focuses on the diffusion mechanism of interface atoms by selecting a more extensive Cu_3_Sn/Cu interface system. A large system generates more crystal defects in the molecular simulation. It is accompanied by the complex mechanism of various crystal defects on diffusion, together with the uncertainty in the location and quantity of crystal defects, which promotes the instability of diffusion. The migration rate of various crystal defects is accelerated in an electric field, affecting grain growth and atomic diffusion at the interface. Thereby, there is no apparent regularity between the diffusion rate and the electric field intensity. However, the diffusion activation energy is equivalent to the potential barrier, which should be overcome by the atom to occupy a new equilibrium position among the adjacent nodes. It is an indicator reflecting the diffusion ability of the atoms. Therefore, the diffusion activation energy Q is employed in this section to analyze the diffusion behavior of the Cu_3_Sn/Cu interface.

**Figure 4:**
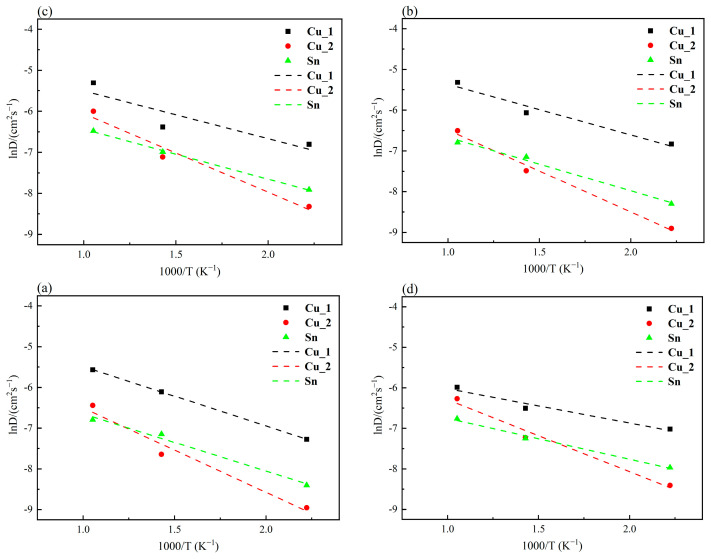
Relationship curves between interface element diffusion coefficient and temperature under different electric field intensities: (**a**) E = 0 V/Å, (**b**) E = 0.002 V/Å, (**c**) E = 0.004 V/Å, and (**d**) E = 0.008 V/Å.

The atomic diffusion coefficient was computed and plotted in Figure 4 with varying electric field intensity for the interfacial atoms at temperatures of 450 K, 700 K, and 950 K. It can be seen that lnD is almost linear with 1000/T. The slope of the fitted line is -−Q/R, which obtains Q of Cu_1, Cu_2, and Sn atoms. The Q of the Cu_2 atom is greater than that of the Cu_1 and Sn atoms, indicating that it is difficult for the Cu_2 atom to overcome an enormous barrier to make the transition.

**Figure 5:**
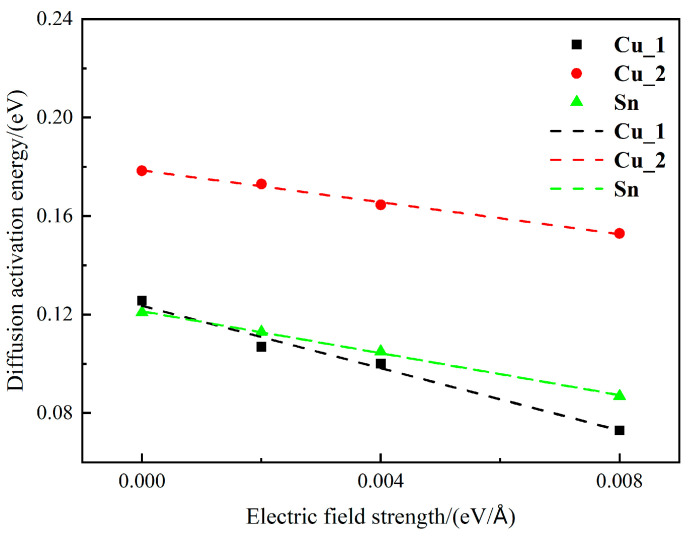
The relationship between different electric field intensities and diffusion activation energy.

The diffusion activation energies Q of Cu_1, Cu_2, and Sn atoms under different electric field intensities are plotted in Figure 5. Q decreases with an increase in electric field intensity. The conclusion is consistent with the conclusion obtained by Cheng [16] through first principles calculations. With the decrease in Q, atoms are more likely to leave their original positions, accelerating the migration rate of various crystal defects. Therefore, it eventually accelerates the diffusion of the interfacial atoms. Similar tendencies are also observed in the literature [12,25,26]. Based on the electron theory, vacancies in a pure metal possess a negative charge, and a lattice defect represents a form of static electrical energy resulting from a thin layer of negative charge that screens positive charges. In the interface system of electrothermal coupling, perturbations in the electric state readily occur at defect sites [26,27]. An external electric field can lower the activation energy of vacancies or boundaries, and the electronegative vacancies diffuse toward the surface and then annihilate. This phenomenon would lead to a concentration gradient of vacancies, which would drive the vacancies to diffuse to the surface. The motion of vacancies is greatly accelerated under the action of the electric field, thus accelerating the diffusion of the Cu_3_Sn/Cu interface.

As shown in Figure 5, the diffusion activation energy of interface atoms is smaller than the calculated value from zero electric field experimental studies [28]. From the analysis of the crystal structure, the interface contains a large number of distortion areas, which will increase the energy of the atoms in this area, making it have a higher vibration frequency. The atoms at the interface are arranged irregularly and have a low density, which also promotes the migration of atoms. Moreover, the interfaces during calculation are all ideally (001) crystal faces, and the incomplete matching of the lattice causes a certain distortion energy between the interfaces, which is conducive to the diffusion of atoms between the interfaces. Since this simulation system is larger, more defects will be generated at the interface, and the migration of crystal defects accelerates the diffusion of interface atoms. In summary, the diffusion activation energy calculated in this study is smaller than the experimental value. But in fact, what we are more concerned about is the changing tendency of the diffusion activation energy from zero electric fields to applying an electric field. As the electric field intensity rises from 0 V/Å to 0.008 V/Å, the diffusion activation energy of Cu_1, Cu_2, and Sn atoms decreases by 41.9%, 14.3%, and 28.1%, respectively. Compared to Cu_2 atoms, the electric field has a more significant impact on the diffusion activation energy of Cu_1 and Sn atoms. The reason is that the bonds and interactions in Cu_3_Sn are more easily broken, and the probability of atomic jumps is more remarkable, which makes the diffusion activation energy of Cu_1 and Sn atoms smaller than that of Cu_2 atoms.

**Figure 6:**
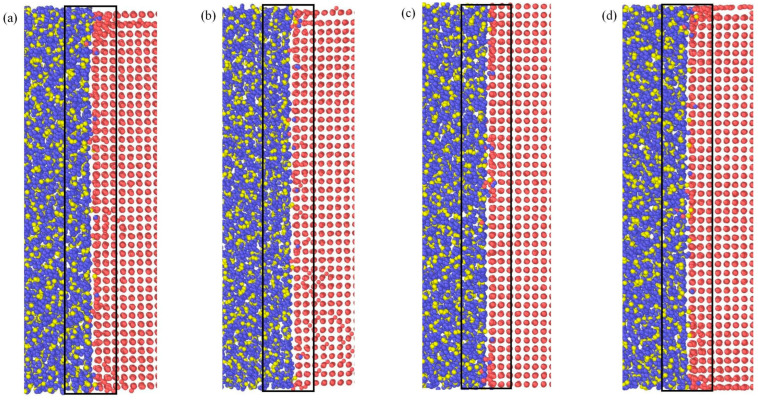
Diffusion of the Cu_3_Sn/Cu interface after 200 ps: (**a**) 0 V/Å, (**b**) 0.002 V/Å, (**c**) 0.004 V/Å, and (**d**) 0.008 V/Å. (The square in Figure 6 indicate the location of diffusion at the Cu_3_Sn/Cu interface).

Figure 6 shows the atomic distributions at the Cu_3_Sn/Cu interface at 200 ps. It can be seen that more atoms in the Cu_3_Sn crystal leave their equilibrium positions and diffuse into the Cu substrate. In contrast, only a few Cu crystal side Cu_2 atoms diffuse into the Cu_3_Sn crystal. In addition, the relative atomic mass of Sn is more significant than that of Cu, and the atomic arrangement in the Cu_3_Sn crystal is relatively loose [18]. Therefore, the crystal defects form easily, and their migration promotes interface diffusion. The migration of the lattice defects is accelerated in an electric field. The Kirkendall effect is more prominent, resulting in decreased reliability of the Cu_3_Sn/Cu interface due to the difference in diffusion rate and atomic diffusion activation energy on both sides of the interface.

### 3.2. Effects of Electrothermal Coupling on the Diffusion Coefficient and Mean Square Displacement

**Figure 7:**
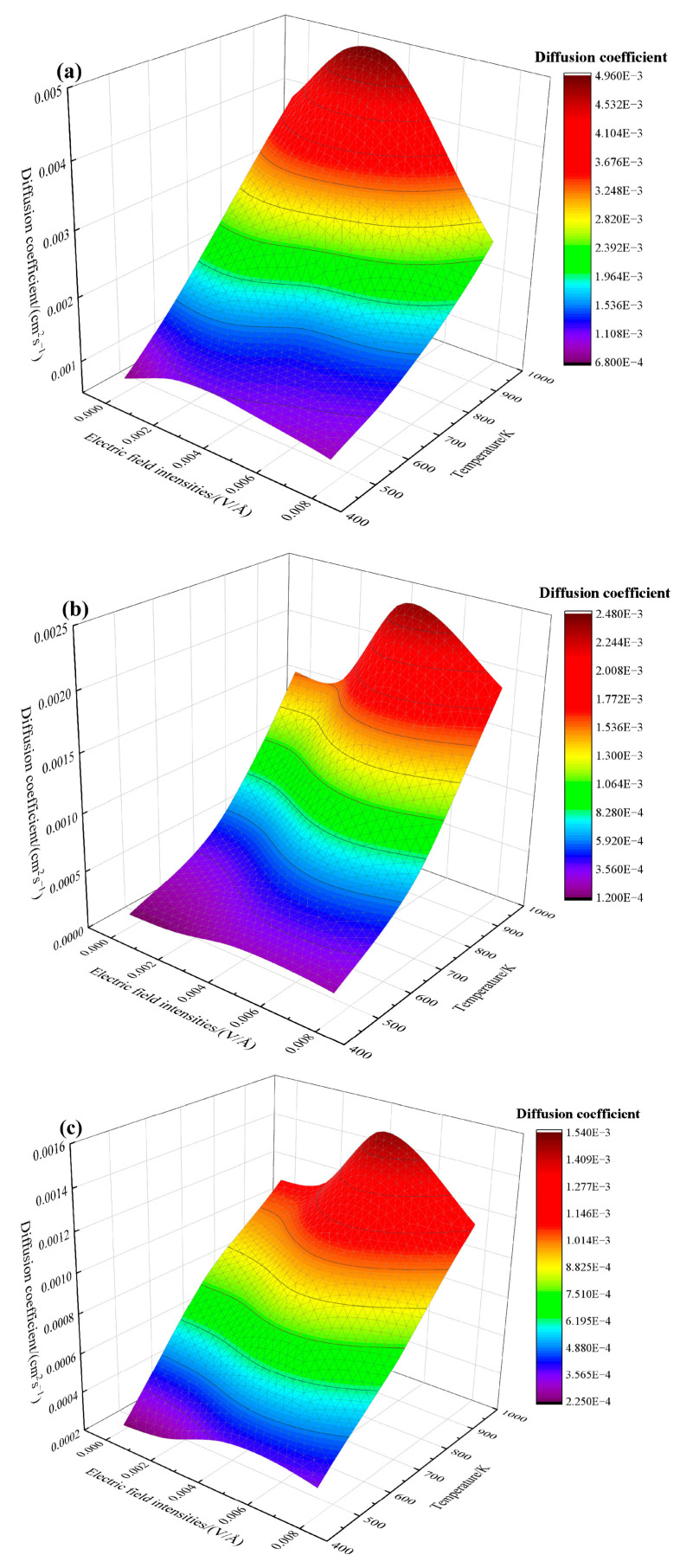
Effect of electrothermal coupling on the diffusion coefficient of atoms at the interface: (**a**) Cu_1, (**b**) Cu_2, and (**c**) Sn.

The three-dimensional diagrams of diffusion coefficient varying with electric field density and temperature are plotted in Figure 7. Electrothermal coupling effects can be obtained. The diagrams of the diffusion coefficient for atoms of Cu_1, Cu_2, and Sn are plotted in Figure 7a–c, respectively. The X-axis is the electric field intensity, and the Y-axis is the temperature. It can be seen that the diffusion coefficients of Cu_1, Cu_2, and Sn atoms increase significantly as the temperature increases. Under the coupling effect of the electric field, the diffusion coefficient reaches its peak value. Specifically, the peak values of Cu_1 atoms are distributed within the electric field intensities of 0.002–0.004 V/Å in the entire temperature range, and those of Cu_2 and Sn atoms are concentrated in the range of 0.004–0.008 V/Å. Furthermore, the contribution of electric field intensity is smaller than that of temperature at 450–800 K. However, the contribution of electric field intensity to the diffusion coefficient increases significantly when the temperature is greater than 800 K.

**Figure 8:**
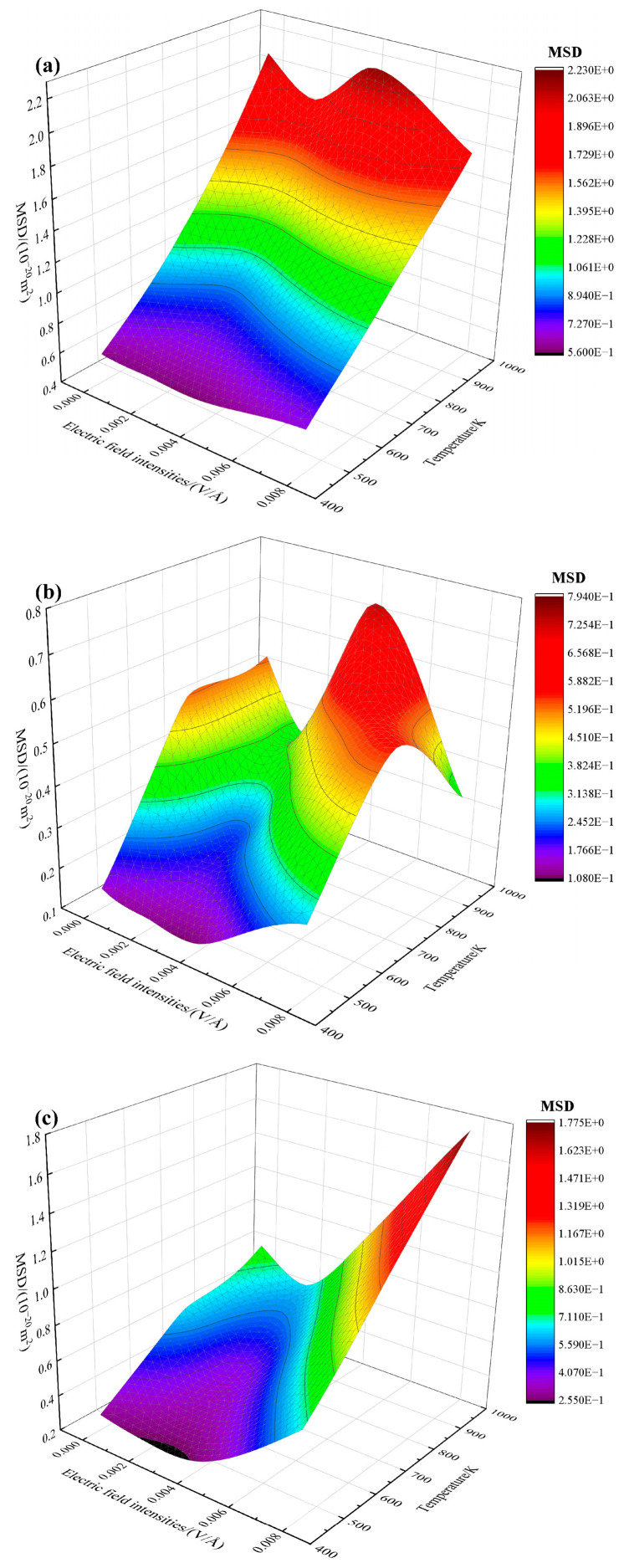
Effect of electrothermal coupling on the MSD value of each atom at the interface. (**a**) Cu_1, (**b**) Cu_2, and (**c**) Sn.

The 3D plots of MSD with varying electric field density and temperature are shown in Figure 8. It can be seen from Figure 8a,c that the MSD values of Cu_1 and Sn atoms increase significantly with temperature compared to those of Cu_2 atoms. At a specific temperature, the MSD of Sn atoms is more sensitive to electric field intensity. MSD of Sn atoms shows a trend of first decreasing and then rising sharply. Electric field intensity and temperature have considerable contributions to the MSD of Sn atoms. In Figure 8b, the MSD of Cu_2 atoms increases first and then decreases with temperature for the electric field intensity within 0–0.002 V/Å and 0.006–0.008 V/Å. Furthermore, the MSD of Cu_2 atoms increases monotonously with temperature for 0.002–0.006 V/Å. The reason is that the increase in temperature has a more substantial effect on the diffusion enhancement of Cu_1 and Sn atoms than that of Cu_2 atoms. Therefore, the diffusion of Cu_1 and Sn atoms around the interface may hinder the diffusion of Cu_2 atoms.

### 3.3. Key Impact Factors in the Cu_3_Sn/Cu Interface Diffusion System under Electrothermal Coupling

In Section 3.1, it has been demonstrated that the acceleration effect of the electric field on the diffusion of the Cu_3_Sn/Cu interface is caused by the decrease in diffusion activation energy *Q*. However, the diffusion mechanism of the Cu_3_Sn/Cu interface is still unknown. Therefore, an investigation of the mechanism of diffusion of the Cu_3_Sn/Cu interface is performed, and a vacancy–response mechanism of diffusion of the Cu_3_Sn/Cu interface is proposed.

The electric field affects the migration of atoms through many factors, namely, electron wind [29], electrostatic force [30], stress gradient [31], temperature gradient [32], and atomic concentration gradient. In the initial stage of electromigration, the atomic concentration gradient of solder joints is minimal. Its impact on electromigration can be ignored in comparison to that caused by the electron wind, temperature gradient, and stress gradient. The atomic concentration gradient in the solder joint continues to increase with electromigration, and its effects on electromigration also increase gradually. It should be noted that the role of the atomic concentration gradient in electromigration needs to be taken seriously.

**Figure 9:**
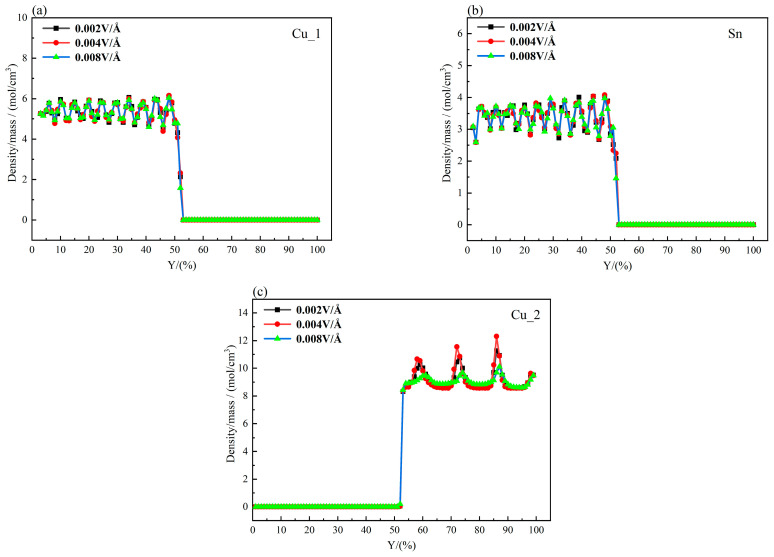
Concentration gradient of each interface atom at 450K: (**a**) Cu_1, (**b**) Sn, and (**c**) Cu_2.

The concentrations of atoms along the Y axis in the Cu_3_Sn/Cu interface at different electric field intensities are plotted in Figure 9a–c for Cu_1, Sn, and Cu_2, respectively. The concentration gradient and diffusion coefficient of Cu_1 atoms have the same trend as follows: $\nabla_{0.008}>\nabla_{0.002}>\nabla_{0.004}$ and $D_{0.008}>D_{0.002}>D_{0.004}$. Similar conclusions can be drawn for the Sn and Cu_2 atoms. The atomic thermal motion is slow at 450 K, and the melting points of Cu_3_Sn and Cu are relatively high. Therefore, it is not enough for the energy of each atom to reach the state of overcoming its diffusion energy barrier. In addition, the concentration variation rate of the Cu_1 atom and Sn atom is negative, and that of the Cu_2 atom is positive, which means that Cu_1, Sn, and Cu_2 atoms diffuse toward the Cu_3_Sn/Cu interface. Under the combined action of the internal driving force of the concentration gradient and the external driving force of the electric field, the probability of an atomic transition increases. When the energy of an atom exceeds the binding energy of surrounding atoms, it will randomly jump to the adjacent equilibrium position. Therefore, in the electrothermally coupled Cu_3_Sn/Cu interface diffusion system, the coupling effects of temperature, electric field, and concentration gradient dominate the diffusion behavior of atoms.

### 3.4. Diffusion Behavior of the Cu_3_Sn/Cu Interface System under Electrothermal Coupling

Regarding the MSD-time curves in Figure 3b,d,f of Cu_1, Cu_2, and Sn atoms at 450 K, the MSD value undergoes an abrupt change at a specific moment. Especially at E = 0.008 V/Å, MSD rises sharply in a very short time, and the transition amplitude is the largest. Then, it remains stable for a relatively long period of time. Concerning E = 0.004 V/Å, the atoms in the interface layer diffuse stably, and the driving forces overcoming the diffusion energy barrier have reached a relatively stable state. The MSD value does not have a transition phenomenon. As to E = 0.002 V/Å, MSD also undergoes a sudden variation lagging behind that of E = 0.008 V/Å, and its transition amplitude is much smaller. The reason is that the diffusion activation energy of E = 0.002 V/Å is more prominent, and the atoms need more energy to overcome the binding energy of the surrounding atoms. The transition stages are indicated with circle symbols in Figure 3. The transition point is related to the electric field application.

**Figure 10:**
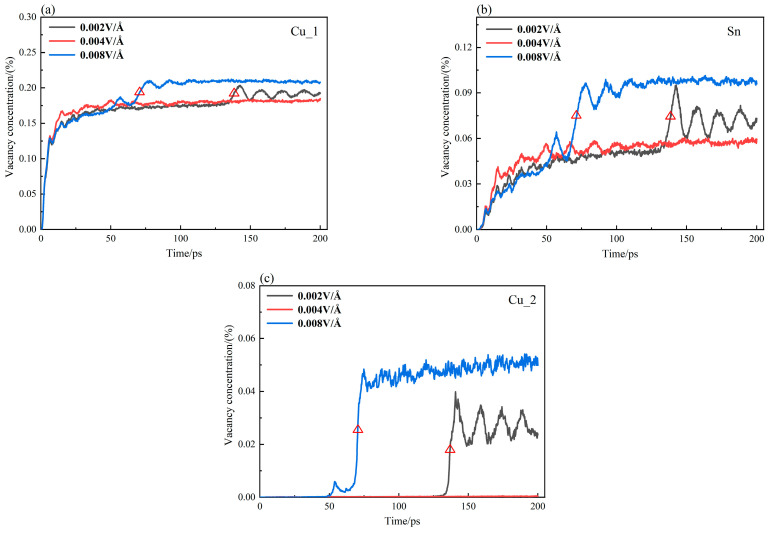
Vacancy concentration-time curve of Cu_1, Sn, and Cu_2 atoms at 450 K. (**a**) Cu_1; (**b**) Sn; (**c**) Cu_2. (The triangle in the figure indicate the mutation phase of the curve).

The vacancy concentrations of Cu_1, Sn, and Cu_2 atoms at 450 K are plotted in Figure 10a–c. In the early stage of diffusion, the vacancy concentration of the atom increases steadily and gradually. After stabilization, the vacancy concentration undergoes a sudden change and reaches stability again. The varying tendency is similar to that in Figure 3b,d,f. This phenomenon becomes more evident under the effect of electrothermal coupling. In terms of the marked parts in Figure 3b,d,f, and Figure 10, the start points of the abrupt change in MSD and vacancy concentration are denoted as $T_{vc}$ and $T_{msd}$, and the endpoints are $T_{vc}^{\prime }$ and $T_{msd}^{\prime }$, respectively. It is found that $T_{vc}$ always lags behind $T_{msd}$, and the difference $\Delta T_{vc}=(T_{vc}^{\prime }-T_{vc})$ and $\Delta T_{msd}=(T_{msd}^{\prime }-T_{msd})$ is approximately equal with an error within 5%, as tabulated in Table 3.

### 3.5. Vacancy Response Mechanism of Cu_3_Sn/Cu Interface Diffusion under Electrothermal Coupling

**Figure 11:**
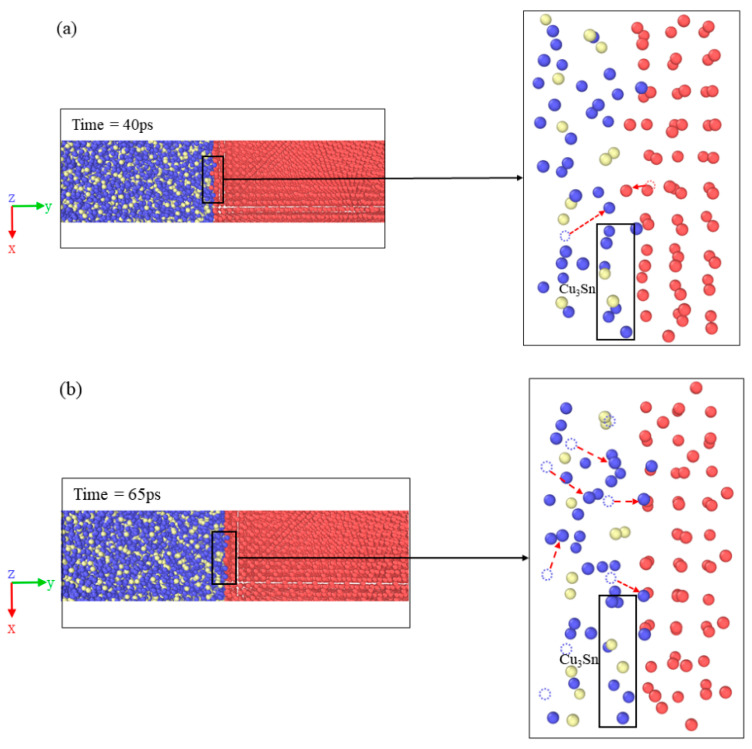

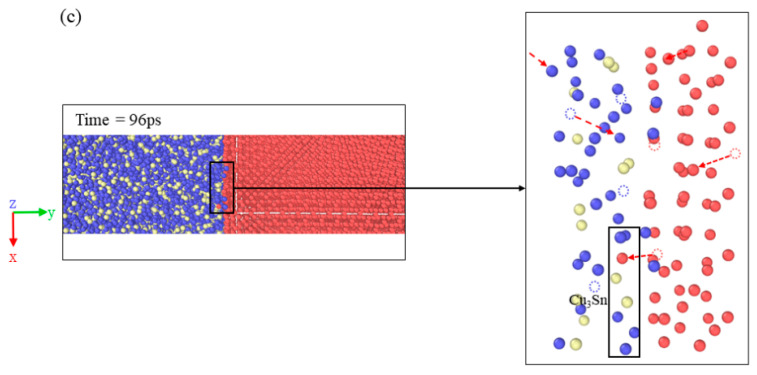
Local atomic displacement, vacancy generation, and atomic transposition process at the interface at 450 K: (**a**) time = 40 ps, (**b**) time = 65 ps, and (**c**) time = 96 ps.

Figure 11 shows the local atomic displacement, generation of vacancies, and the atomic transposition process at 450 K. In Figure 11a, a few Cu_1 and Cu_2 atoms deviate from the equilibrium position at 40 ps (40 ps is smaller than $T_{vc}$), and the diffusion tends to be stable. The energy of atoms at the interface accumulates rapidly in the electric field. In Figure 11b, a considerable number of Cu_1 atoms migrate at 65 ps; thereby, vacancies are generated, and the surrounding atoms exhibit a tendency to relax. As a result, the volume occupied by a vacancy $\Omega_{v}$ becomes a fraction of an atomic volume $\Omega_{a}$. The accumulation of vacancies can result in localized volume shrinkage [33]. In the process of the motion of vacancies, vacancy–solute atom pairs might be formed, which plays a significant role in diffusion at a relatively low temperature. Compared with time = 40 ps, the local atomic distribution changes considerably at time = 65 ps, and the vacancy accelerates to migrate to the interface or surface. At this stage, interfacial diffusion is the strongest. When time = 96 ps, according to Figure 3 and Figure 10, the MSD value increases linearly, and the diffusion coefficient is almost constant. The vacancy concentration shows a steady trend, indicating that interfacial diffusion has reached a stable state and the internal vacancy diffusion mechanism at the interface has reached a balance.

Figure 11c shows the atomic transposition process at 90 ps. Cu_1 and Cu_2 atoms migrate through vacancies in the lattice. Compared to 65 ps, the local atomic distribution varies slightly, and the interfacial diffusion is stable. It is worth noting that from 40 ps to 96 ps, the displacement of Sn atoms is minimal. It is found by OVITO observation that most Sn atoms oscillate around their original position without leaving their original equilibrium position. This is because the formation energy of a vacancy in the Sn atom is E_f_ = 1.24 eV, which is much greater than that of the Cu_1 and Cu_2 atoms [34,35]. Meanwhile, the atomic radius and relative mass of Sn in the Cu_3_Sn lattice are larger than those of the Cu_1 atom. Therefore, the activation energy of Sn atoms is greater than that of Cu.

A complete Cu_3_Sn structure can be observed in Figure 11a. When the time is longer than $T_{vc}$, Cu_1 atoms in the Cu_3_Sn structure overcome the free barrier energy, provide the energy to form vacancies, and gradually leave their equilibrium position. Comparing Figure 11a,b, it can be seen that two Cu atoms in Cu_3_Sn tend to the position where the vacancy is generated, and the Cu_3_Sn structure is no longer stable. Until 96 ps, as shown in Figure 11c, the Cu_1 atoms break entirely away from the Cu_3_Sn structure, and the Cu_2 atoms in the Cu substrate migrate to the vacancies to reach a new equilibrium state. The repeating process of the generation and occupation of vacancies dramatically accelerates the Cu_3_Sn/Cu interfacial diffusion.

A vacancy response mechanism for the Cu_3_Sn/Cu interface was proposed based on the aforementioned discussion. During the initial phase of interfacial diffusion, atomic energy rapidly accrues due to the action of the temperature field, concentration gradient, and external electric field. Some atoms deviate from their original positions and generate a large number of vacancies. The variation of vacancy leads to an increase in mixing entropy and vibration entropy induced by atomic vibration, which keeps the crystal in a higher energy state and intensifies the diffusion. Therefore, MSD and vacancy concentrations rise rapidly in this process. The interfacial diffusion gradually tends to be stable in simulation. Much literature [18,36] depicts that the MSD value and diffusion coefficient are stable without applying an electric field. The electric field not only drives the diffusion of atoms but also accelerates the migration rate of various crystal defects and the migration of vacancies, which drives the internal vacancies to diffuse to the interface or surface. The interfacial diffusion is enhanced by the electric field. The local atomic transposition mechanism shown in Figure 11 provides evidence of the discussion.

It can be concluded that the electric field does not have direct effects on vacancies. The vacancy concentration increases as the atom leaves the equilibrium position, which is reflected by the lag of the abrupt change in the vacancy concentration behind that of the MSD value. The vacancy response to MSD is also the response of vacancies to the electric field. In terms of diffusion activation energy, atoms are more likely to migrate from one equilibrium position to another with increased electric field intensities. Vacancy concentration increases, and the surrounding atoms tend to relax around the vacancies. Vacancy motion is greatly accelerated under the action of the electric field, thereby generating more crystal defects and finally enhancing interfacial diffusion. Then, MSD and vacancy concentration reach a new stable state with some minor oscillations locally. The oscillation is due to the electric field enhancing the interface perturbation, while the interfacial diffusion suppresses the interface instability. Therefore, the oscillation is generated by the competition of interface disturbance and interface diffusion.

## 4. Discussion

Electronic devices are progressing towards miniaturization, increased integration, and expanded functionality. The increase in electric field intensity causes electromigration problems in interconnect solder joints, affecting the long-term reliability of device operation. The diffusion mechanism of solder joints under the action of electrothermal coupling provides guidance and reference for the packaging design of the high-density interconnection and the high-power density device.

On the one hand, the diffusion activation energy decreases with the increase in electric field intensities, accelerating the diffusion of the Cu_3_Sn/Cu interface. This is particularly evident in miniaturized microbump interconnects and high-power-density semiconductor interconnects. When designing the microsolder joints, the size and number of the microsolder joints need to be considered to ensure reasonable electric field intensity at the interface to avoid causing a serious Kirkendall effect and ensure the reliability of the electronic assemblies.

On the other hand, in the diffusion system of the Cu_3_Sn/Cu interface under the action of electrothermal coupling, temperature, electric field, and concentration gradient are the factors that dominate the diffusion behavior. It emphasizes the complexity of the interaction of multiple factors. Furthermore, at higher temperatures, the contribution of electric field intensity to the diffusion coefficient will increase significantly, which will further affect the thermal stability of the material. Therefore, the electric field intensity and temperature distribution of the interconnect solder layer should be controlled simultaneously during package design, thereby improving the quality and long-term reliability of the solder joint interface. Possible strategies include optimizing heat dissipation, avoiding local electric field concentrations, etc.

## 5. Conclusions

The LAMMPS package was employed to simulate the atomic diffusion behavior at the Cu_3_Sn/Cu interface. The diffusion activation energy of atoms at the interface was calculated under various temperatures and electric field intensities. The relationship between MSD, vacancy concentration, and electric field intensities was investigated. The following conclusions were drawn, combined with the local vacancy transposition mechanism:

1. The diffusion activation energy decreases with the increase in electric field intensities, accelerating the diffusion of the Cu_3_Sn/Cu interface. The diffusion activation energy of the Cu_2 atoms in the Cu pad is greater than that of the Cu_1 and Sn atoms in Cu_3_Sn, indicating that Cu_2 atoms must overcome a more significant diffusion energy barrier to migrate.
2. In the diffusion system of the Cu_3_Sn/Cu interface under the action of electrothermal coupling, the influence of the concentration gradient cannot be ignored. Temperature, electric field, and concentration gradient dominate the diffusion behavior.
3. The contribution of electric field intensity to the diffusion coefficient increases significantly when the temperature is greater than 800 K. The increase in temperature has a more substantial effect on the diffusion enhancement of Cu_1 and Sn atoms than that of Cu_2 atoms. The diffusion of Cu_1 and Sn atoms around the interface may hinder the diffusion of Cu_2 atoms.
4. The vacancy response mechanism of the Cu_3_Sn/Cu interface under electrothermal coupling was proposed. The electric field has direct effects on the atoms. The migration of vacancies is accelerated by the increased vacancy content as more atoms leave the equilibrium position. The lag in the abrupt variation of the vacancy concentration behind that of MSD reflects the response of vacancies to the electric field.

This study provides a reference for further exploration of the diffusion mechanism of the Cu_3_Sn/Cu interface and improves the reliability of the high-density microelectronic package interface.

In terms of the electromigration problem, which is caused by the effects of thermal–mechanical–electrical multifield coupling, the electromigration failure phenomenon is found to be due to the simultaneous action of multiple migration driving modes such as electromigration, thermal migration, stress migration, and chemical migration. Additionally, solder joints may be subjected to assembly stress during service. The effect of stress migration in solder joints on interfacial diffusion behavior has not been considered in this study. Therefore, the diffusion behavior of Cu and Sn under thermal–mechanical–electrical coupling should be further investigated, and the interactions between these factors and their effects on the diffusion behavior also need to be revealed.

## Figures and Tables

**Table 1:** MEAM potential parameters used in LAMMPS.

Element	$E_{c}(eV)$	A	*R*_0_ (Å)	α	$\beta^{\left(0\right)}$	$\beta^{\left(1\right)}$	$\beta^{\left(2\right)}$	$\beta^{\left(3\right)}$	$t^{\left(1\right)}$	$t^{\left(2\right)}$	$t^{\left(3\right)}$	$\rho_{0}$	$i_{bar}$
Cu	3.62	1.07	2.50	5.11	3.62	2.20	6.00	2.20	3.14	2.49	2.95	1.00	0
Sn	3.08	1.00	3.44	6.20	6.20	6.00	6.00	6.00	4.50	6.50	−0.18	1.00	0
Cu_3_Sn	3.50		2.68	5.38									

**Table 2:** Values of screening parameters, $C_{ijk}$. Note that one refers to Cu atoms and two to Sn atoms.

	*ijk*					
	111	112	212	121	221	222
$C_{\max}$	2.8	2.8	2.8	2.8	2.8	2.8
$C_{\min}$	2.0	0.8	0.8	0.8	0.8	0.8

**Table 3:** $\Delta T_{msd}$ and $\Delta T_{msd}$ values of each atom at 450 K and their error rates.

Electric Field Intensity (V/Å)	Cu_1			Cu_2			Sn		
	$\Delta T_{msd}$	$\Delta T_{vc}$	err (%)	$\Delta T_{msd}$	$\Delta T_{vc}$	err (%)	$\Delta T_{msd}$	$\Delta T_{vc}$	err (%)
0.002	9.514	9.901	4.07	9.987	9.557	4.31	11.925	11.537	3.25
0.008	11.537	11.925	3.36	24.548	25.141	2.42	13.560	12.700	6.35

## Data Availability

Data are available on request.

## Nomenclature

$A_{i}$	the structural parameter
$C_{\max}$	the maximum values of screening parameters
$C_{\min}$	the minimum values of screening parameters
D	the diffusion coefficient
E	the electric field intensity
$E_{i}^{0}$	the binding energy of atom *i*
$E_{c}$	cohesive energy of the reference structure for the I-J mixture, eV
$E_{total}$	the total energy of an atomic system
$E_{f}$	the formation energy of a vacancy
err	$\frac{\left|\Delta T_{msd}\;-\;\Delta T_{vc}\right|}{\Delta T_{msd}}$
$F_{i}$	the embedding energy of the *i*th atom
$f_{j}$	the electron density of atom *j*
IMC	intermetallic compounds
$i_{bar}$	the parameter selects the form of the function G(Gamma) used to compute the electron density
MD	molecular dynamics
MEAM	the modified embedded atom method
MSD	mean square displacement
NVT	canonical ensemble
Q	the diffusion activation energy
R	the Boltzmann constant, JK^−1^
$R_{ij}$	the distance between atom *i* and atom *j*
$R_{0}$	the equilibrium nearest-neighbor distance
$r_{c}$	the cutoff radius
$r_{i}(0)$	the initial position of the atom at the initial time
$r_{t}(0)$	the position of atom *i* at time t
r(t)	the position of the atom at time *t*
T	temperature, K
$T_{vc}$	the start points of the abrupt change in vacancy concentration
$T_{msd}$	the start points of the abrupt change in MSD
$T_{vc}^{\prime }$	the endpoints of the abrupt change in vacancy concentration
$T_{msd}^{\prime }$	the endpoints of the abrupt change in MSD
$\Delta T_{vc}$	time span of the abrupt change in vacancy concentration
$\Delta T_{msd}$	time span of the abrupt change in MSD
v(t)	the velocity of the atom at time t
$\phi_{ij}$	the two-body interaction potential between atom *i* and atom *j*
$\alpha_{j}^{1}$	the constants related to atom *j*
$\alpha_{j}^{2}$	the constants related to atom *j*
$\alpha_{k}^{1}$	the constants related to atom *k*
$\alpha_{k}^{2}$	the constants related to atom *k*
$\theta_{ijk}$	the angle between atom *i*, *j*, and *k* when atom *i* is at the center
ϕ(r)	the general equation of the two-body potential
α	the exponential decay factor for the universal energy function
$\beta^{\left(0\right)},\beta^{\left(1\right)},\beta^{\left(2\right)},\beta^{\left(3\right)}$	the exponential decay factors for the atomic densities
$t^{\left(1\right)},t^{\left(2\right)},t^{\left(3\right)}$	the weighting factors for the atomic densities
$\rho_{i}$	the electron density
∇	the concentration gradient
$\Omega_{v}$	the volume occupied by a vacancy
$\Omega_{a}$	a fraction of an atomic volume
δt	the time step
*v*	vacancy
*a*	atom
*vc*	vacancy concentration
*msd*	mean square displacement
